# An App-Based Intervention to Support First Responders and Essential Workers During the COVID-19 Pandemic: Needs Assessment and Mixed Methods Implementation Study

**DOI:** 10.2196/26573

**Published:** 2021-05-20

**Authors:** Stacie Vilendrer, Alexis Amano, Cati G Brown Johnson, Marissa Favet, Nadia Safaeinili, Jacqueline Villasenor, Jonathan G Shaw, Attila J Hertelendy, Steven M Asch, Megan Mahoney

**Affiliations:** 1 Division of Primary Care and Population Health Stanford University School of Medicine Stanford, CA United States; 2 College of Liberal Arts and Sciences University of Iowa Iowa City, IA United States; 3 Health Policy and Management University of California, Berkeley Berkeley, CA United States; 4 John Muir College University of California, San Diego La Jolla, CA United States; 5 Department of Information Systems and Business Analytics College of Business Florida International University Miami, FL United States; 6 Disaster Medicine Fellowship Department of Emergency Medicine Beth Israel Deaconess Medical Center Boston, MA United States; 7 Center for Innovation to Implementation Veterans Affairs Menlo Park, CA United States

**Keywords:** COVID-19, pandemic, health literacy, social media, quality improvement, police, emergency responders, physicians, disasters, natural disasters, health behavior, literacy, app, intervention, adoption, accessibility, usability, support, testing

## Abstract

**Background:**

The COVID-19 pandemic has created unprecedented challenges for first responders (eg, police, fire, and emergency medical services) and nonmedical essential workers (eg, workers in food, transportation, and other industries). Health systems may be uniquely suited to support these workers given their medical expertise, and mobile apps can reach local communities despite social distancing requirements. Formal evaluation of real-world mobile appbased interventions is lacking.

**Objective:**

We aimed to evaluate the adoption, acceptability, and appropriateness of an academic medical centersponsored app-based intervention (COVID-19 Guide App) designed to support access of first responders and essential workers to COVID-19 information and testing services. We also sought to better understand the COVID-19related needs of these workers early in the pandemic.

**Methods:**

To understand overall community adoption, views and download data of the COVID-19 Guide App were described. To understand the adoption, appropriateness, and acceptability of the app and the unmet needs of workers, semistructured qualitative interviews were conducted by telephone, by video, and in person with first responders and essential workers in the San Francisco Bay Area who were recruited through purposive, convenience, and snowball sampling. Interview transcripts and field notes were qualitatively analyzed and presented using an implementation outcomes framework.

**Results:**

From its launch in April 2020 to September 2020, the app received 8262 views from unique devices and 6640 downloads (80.4% conversion rate, 0.61% adoption rate across the Bay Area). App acceptability was mixed among the 17 first responders interviewed and high among the 10 essential workers interviewed. Select themes included the need for personalized and accurate information, access to testing, and securing personal safety. First responders faced additional challenges related to interprofessional coordination and a culture of heroism that could both protect against and exacerbate health vulnerability.

**Conclusions:**

First responders and essential workers both reported challenges related to obtaining accurate information, testing services, and other resources. A mobile app intervention has the potential to combat these challenges through the provision of disease-specific information and access to testing services but may be most effective if delivered as part of a larger ecosystem of support. Differentiated interventions that acknowledge and address the divergent needs between first responders and nonfirst responder essential workers may optimize acceptance and adoption.

## Introduction

The COVID-19 pandemic has presented unique obstacles to first responders as well as to other essential workers who ensure that health and other basic needs of the public continue to be met [1,2]. Health systems may be uniquely positioned to support these individuals given their expertise and position embedded within local communities. Efforts to understand the challenges of first responders and essential workers and to evaluate support of these groups by health systems during a pandemic response are needed.

The definition of a first responder varies between state and federal agencies but commonly encompasses emergency medical personnel, firefighters, and law enforcement officers [3]. Their duties can lead to adverse mental, physical, and social consequences [4]. Although academic attention has focused on the intense mental and emotional strain on health workers, such as during the severe acute respiratory syndrome (SARS) epidemic [5,6] and the COVID-19 pandemic [7-9], nonmedical first responders also face adverse mental health consequences [10,11]. Other adverse effects include worker absenteeism [12,13], an exacerbation of predisaster socioeconomic inequalities [14], and an increased risk of contracting the disease itself in an outbreak setting [15,16].

Less is known about the impact of disasters on essential workers not otherwise considered to be first responders, defined by their work in food and agriculture, construction, transportation, and other sectors of the economy deemed essential [17,18]. COVID-19 has spurred research in this population, with documented increased rates of depression and anxiety [19] and substance abuse [20] during the pandemic. Most concerning, the relative economic vulnerability of essential workers, including their need to leave home to work, further exacerbates existing socioeconomic and racial disparities seen in COVID-19 morbidity and mortality [17,21-23].

Certain factors can mitigate the stress experienced by first responders and essential workers [20]. Knowledge about a disease process may be protective, as essential workers without a health care background appear to experience a higher level of distress than workers who have training in disease processes [24]. Open, reliable information channelseven in a setting with many unknownsmay also be protective [25]. To date, documented efforts to support these workers have focused on prioritized access to testing [26], mental health support [18], and other health resources [27,28], but not necessarily on access to information.

Dissemination of this information occurs through formal and informal networks during a pandemic response [29]. However, the challenges brought on by the COVID-19 pandemic have highlighted a pre-existing disconnect between the health system and public health infrastructure [30,31]. Without these partnerships, first responders and essential workers cannot benefit from the expertise of the health systems located right in their communities. Given the uncertainties inherent in an unfolding pandemic, additional channels that draw upon health system expertise may be needed.

To this end, digital technologies, including mobile apps, appear to provide unique support in the context of social distancing efforts during a pandemic [32-34]. Formal evaluations of digital efforts to support first responders and essential workers are lacking; however, optimizing these upstream interventions may help mitigate health disparities and other adverse effects associated with their roles during a pandemic.

## Methods

We conducted a qualitative user needs assessment and implementation evaluation (outcomes including adoption, appropriateness, and acceptability) [35] of an academic medical centersponsored app-based intervention designed to support the information and testing needs of first responders and essential workers early in the COVID-19 pandemic. We further used app download data to triangulate [36] our understanding of the adoption of the app among the target populations using a convergent [37] mixed methods approach.

### Setting

In April 2020, a quaternary academic medical center, Stanford Health Care (SHC, Palo Alto, California, United States) in partnership with a large technology company (Apple, Cupertino, California, United States) launched the *Stanford Medicine COVID-19 Guide for First Responders and Essential Workers* app (COVID-19 Guide App) with the goal of providing evidence-based information related to COVID-19, screening guidance, and access to testing services to support 1.1 million first responders and essential workers in the San Francisco Bay Area [38-40]. The dissemination effort by the institution included emails to local first responder and essential worker representatives, facilitated in part through previously established partnerships between SHC emergency physicians and emergency medical services (EMS) and fire organizations within the community developed through the institutions EMS Fellowship. This study was conducted by an interdisciplinary evaluation group led by the Stanford Medicine Evaluation Sciences Unit. First responder interview participants were recruited from Bay Area county police, fire, and EMS departments and among physicians connected to the health systems EMS physician fellowship. Essential worker interview participants were recruited from local grocery stores, gas stations, care facilities, and civil infrastructure support businesses.

### Intervention

**Figure 1:**
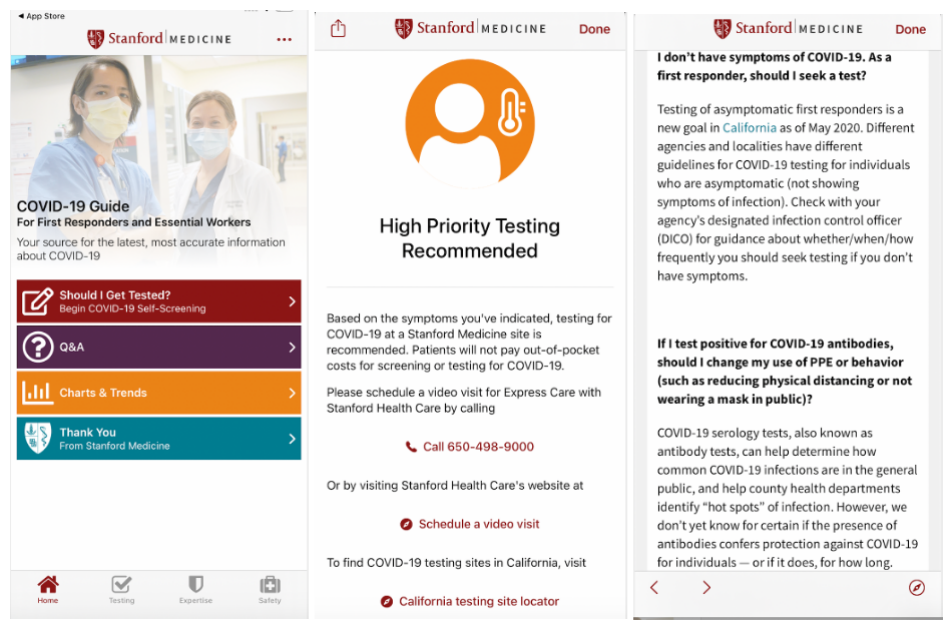
Example user displays of the Stanford Medicine COVID-19 Guide for First Responders and Essential Workers app.

The COVID-19 Guide App includes a screening questionnaire that provides testing recommendations with links to access further testing and care, COVID-19 resources from Stanford Health Care physicians and scientists, and foundational information on viruses and prevention (Figure 1) [38].

### Sample

First responder participants were drawn from police, fire, and EMS departments in Alameda, San Mateo, San Francisco, and Santa Clara counties through email and telephone call outreach to local departments. All fire personnel are cross-trained in EMS, although not all EMS personnel have fire training [41]; participants from both groups were recruited. Emergency medicine physicians who partnered with local fire and police departments through the EMS Fellowship [42] at Stanford School of Medicine were also interviewed using a convenience sampling approach. Snowball sampling was leveraged across all categories to connect with additional participants in harder-to-reach groups. Interviews were conducted until thematic saturation within each professional subgroup was reached.

Essential workers were recruited using a convenience sampling approach in which researchers sought interviews in person at local businesses. When a paucity of Spanish-speaking essential workers was recognized, we shifted to a telephone-based convenience sampling approach targeted at this population to better reflect demographics of the region [43] until thematic saturation for both English- and Spanish-speaking essential workers was reached. Pre-existing experience with the app was not a requirement for participation, and the interview protocol itself accommodated both prior experience and no prior experience with the app (Multimedia Appendix 1).

### Qualitative Data Collection

From May to September 2020, 30-minute semistructured interviews using a protocol grounded in the Consolidated Framework for Implementation Research were conducted by multiple authors (SV, NS, AA, MF, CBJ) (Multimedia Appendix 1) [44]. Interviews were conducted with two or more researchers present, with rare exceptions for note-taking, training, and analytic purposes. First responder interviews were conducted by telephone and videoconference; essential worker interviews were conducted by telephone and in person. For Spanish speakers, a professional interpreter was enlisted to interpret interviewer questions and interviewee responses. First responder interviews were tracked confidentially, recorded, and transcribed verbatim using Rev [45]. Essential worker interviews were not recorded to protect the anonymity of these more vulnerable interviewees in their work-based settings. For these interviews, near-verbatim field notes were taken, and two or more researchers who were present at each interview contributed to the field notes in a debrief discussion immediately following the interview.

### Quantitative Data Collection

Descriptive quantitative data, including the total number of downloads at the conclusion of the evaluation on September 1, 2020, were drawn from the app administrator account. User characteristics, including demographic and use data, were not available due to restrictions in data use agreements.

### Qualitative Analysis

Interview transcripts were analyzed using an inductive rapid analytic process in which two researchers coded all interviews (SV, AA) [46,47]. We used an inductive-deductive approach to identify a priori themes related to access to information and testing services and emergent themes. Excerpts were presented in a matrix format following rapid analytic procedure methodology [48]. The matrix was populated with notes and quotations (AA) and reviewed by qualitative team members (SV, AA, MF) to reach consensus and compare themes across worker subgroups [49]. The Implementation Outcomes Framework guided the evaluation and presentation of implementation outcomes (appropriateness, acceptability, and adoption) [50].

Throughout the assessment, early findings were reported back to operational leads to inform ongoing improvement using the Lightning Report Method described in 2019 by Brown-Johnson et al [51]. This project was reviewed by the Stanford Institutional Review Board and did not qualify as human subjects research (Protocol ID 56126). All participants gave verbal consent to be interviewed, and first responders gave consent to be audio-recorded.

## Results

### Participant Characteristics

A total of 27 interviews were conducted with 17 first responders (63%) and 10 essential workers (37%) (Table 1). The number of participants within each subcategory was comparable, including 5 police (29%), 7 emergency medical technician (EMT) (41%), 4 EMT with fire training (EMT/Fire, 59%), and 5 physician police/EMS advisor (PoliceMD/FireMD, 29%) interviewees in the group of 17 first responders and 5 food and agriculture (50%) and 5 transportation and civil infrastructure (50%) interviewees in the group of 10 essential workers. The food and agriculture subgroup included 4 grocery workers and 1 dining supervisor, while the transportation and civil infrastructure subgroup included 3 gas attendants, 1 bus driver, and 1 electrician. First responders tended to be male (16/17, 88%), 30-49 years old (11/17, 65%), and White (10/17, 59%), and most reported working in their field for more than 10 years (10/17, 59%). The essential workers were 50% male (5/10), of varying ages, mostly Hispanic/Latino (7/10, 70%), and had worked in the field for either less than 5 years (4/10, 40%) or more than 10 years (5/10, 50). The primary language of half of the essential workers (5/10, 50%) was Spanish, whereas all first responder interviews were conducted in English.

**Table 1:** Participant demographics of the first responders and essential workers (N=27).

Variable		Value, n (%)	
		First responders (n=17)	Essential workers (n=10)
**Role**			
	Police	5 (29)	N/A^a^
	EMS^b^ overall	7 (41)	N/A
	EMS with fire training	4 (59)	N/A
	Physician police/EMS advisor	5 (29)	N/A
	Food and agriculture	N/A	5 (50)
	Transportation and civil infrastructure	N/A	5 (50)
**Gender**			
	Male	15 (88)	5 (50)
	Female	2 (12)	5 (50)
**Age (years)**			
	20-29	2 (12)	1 (10)
	30-39	7 (41)	2 (20)
	40-49	4 (24)	2 (20)
	50-59	3 (18)	1 (10)
	60-69	1 (6)	3 (30)
	70-79	0 (0)	1 (10)
**Years working in occupation**			
	0-4	1 (6)	4 (40)
	5-9	6 (35)	1 (10)
	10+	10 (59)	5 (50)
**Race/ethnicity**			
	Hispanic/Latino	5 (29)	7 (70)
	American Indian or Alaskan Native	0 (0)	0 (0)
	Asian or Asian American	2 (12)	1 (10)
	Black or African American	0 (0)	0 (0)
	Native Hawaiian or other Pacific Islander	0 (0)	0 (0)
	White	10 (59)	0 (0)
	Other/multiracial	0 (0)	2 (20)
**Primary language**			
	English	17 (100)	5 (50)
	Spanish	N/A	5 (50)

^a^N/A: not applicable.

^b^EMS: emergency medical services.

### Implementation Outcomes of the COVID-19 Guide App

Implementation outcomes of the COVID-19 Guide App differed between the first responder and essential worker groups. Perspectives on the app varied by group, with moderate to low adoption, mixed appropriateness, and mixed acceptability seen in first responders, and low adoption, high appropriateness, and high acceptability seen in essential workers.

#### Adoption

The COVID-19 Guide App received 8262 views from unique devices, from which 6640 individuals downloaded the app (80.4% conversion rate) from the launch date of April 7, 2020 to the end of the evaluation period on September 1, 2020. Assuming all downloads were in the target demographic within the Bay Area, this suggests a 0.61% adoption rate of the regions 1,093,781 first responders and essential workers [39,40].

In the qualitative interviews, 2 participants had used the app and were able to share their experience directly. The majority were not familiar with the app but shared their perspectives based on a short description of the app.

Moderate to low adoption was described in the first responder group. First responder interviews shed light on the underlying reasons limiting widespread adoption. Physicians largely felt their involvement in the app development occurred fairly late in the game (FireMD 1, 2), perhaps due to the poor visibility of the emergency fellowship program within the institution (FireMD 1, 4).

In the essential worker group, on the other hand, none of the interviewees reported direct experience with the app, although their attitudes toward a theoretical app that provided COVID-19 testing were positive (see the *Acceptability* section below).

#### Appropriateness

First responders reported mixed perceptions of the appropriateness of the COVID-19 Guide App. All interviewees reported owning smartphones, with most using mobile apps for work. Despite the seeming appropriateness of an app-based solution for this group, some reported that the features of this app were less relevant for first responders. Although some thought the screening features and link to testing resources were personally helpful (PoliceMD 3, EMT/Fire 2) or useful for patients (EMT/Fire 7), others felt these features were already covered by other processes in their organization. Some reported frustration with the apps inability to link a positive symptom screen to a scheduled test due to technical and privacy limitations; instead, the app would take [them] to the end of a little pathway that would be like, You should call your [department infection control officer] to get tested. No [kidding]. (FireMD 2).

Conversely, essential workers reported high appropriateness of the COVID-19 Guide App. Similar to first responders, all essential workers owned smart phones, and an overwhelming majority used mobile applications for work, indicating an ability to access and navigate mobile applications.

#### Acceptability

First responders expressed mixed acceptability of the app. A few participants who had directly used the app had positive perceptions (EMT/Fire 3, Police 1), sharing benefits of quick answers that were super easy to access (EMT/Fire 3).

Additionally, first responders also noted the need for a succinct, trusted data source (EMT/Fire 2), such as this app, particularly one that agreed with county information (Police 3, FireMD 4, PoliceMD 1). Indeed, brevity and trusted branding was seen as highly valuable and missing after a midimplementation shift to incorporate an outside partnership to produce content: Now it's just too hard to filter what is coming as sort of medical recommendation versus what almost seemed like an ad (FireMD 4).

Although no essential workers reported direct experience with the app, their attitudes toward a theoretical app that provided COVID-19 testing and disease information were overwhelmingly positive: Im looking forward to this app (Grocery 1).

Like first responders, essential workers reported a desire for succinct, up-to-date, and trustworthy information (Grocery 4, Food Services 5). Essential workers noted that ideally, information would be presented in multiple languages to address reported widespread misinformation, particularly in nonEnglish-speaking communities (Gas 2, Electrician 4). This information would also ideally be delivered through social media channels by physicians or nurses, as trust in the health system was reportedly higher than in media or government.

### Challenges Faced by First Responders and Essential Workers

First responders and essential workers experienced a number of related challenges, described in the following qualitative themes: a need for personalized information with no clear playbook, parsing information takes time, misinformed beliefs fuel conflict, priority testing gets them back to work, fear of bringing an amorphous enemy home, and a lack of resources to fight the pandemic. First responders faced additional challenges related to the need for interprofessional coordination and a double-edged culture of heroism that could both protect against and exacerbate mental and health vulnerabilities (Table 2).

**Table 2:** Impact of the COVID-19 pandemic on first responders and essential workers.

Challenges	Illustrative quotations	
	First responders	Nonfirst responder essential workers
Need for personalized answers with no clear playbook	When COVID hit, I was taken out of my primary role and put into a COVID response detail (Police 1) And early on it was a lot of questions [from EMS^a^] this patient didn't tell us she had a fever but when they got to the hospital, she had a fever and she turned out to have been on the Princess Cruise [early COVID-19 outbreak] it was tough because there wasn't a clear playbook for so many different situations. (Fire MD 4)	Before, food was served in a dining roomnow residents eat in their own rooms. We set up dining carts to bring food to residents, which is exhausting. [paraphrased] (Food Services 5) I want information in Spanish (Gas 2) I think it would be helpful to have more knowledge on herbal treatments for COVID I prefer to use this (Electrician 4)
Parsing information takes time	I pretty much rely on my division chief to disseminate information, because he spends all day reading and looking at case studies and such. (EMT^b^/Fire 7)	We are being bombed by so much information everywhere, so it is hard to know what is true. (Bus Driver 5)
Misinformed beliefs fuel conflict	The amount of misinformation has been pretty surprising. So, I dont know that our fire departments are affected more heavily than anyone else, but theres a lot of suspicion and a lot of misinformation. (FireMD 2) We've had a couple people who have come up and said, COVID-19 is a conspiracy. It's not real.. just acting as though we're propagating this lie, which, as firefighters, we're not used to [such] conflict with our community membersIt's a little bit disappointing to see people, A: deny it and B: be confrontational with us. (EMT/Fire 3)	We have to deal with [people not complying with mask policies] every single day A lot of people have the mask on but around their neck (Bus Driver 1) I dont trust information from the government. I dont think it is accurate or reliable. The number of sick people is overexaggerated they are trying to scare people. (Food Services 5) A German doctor is leading the work around chlorine dioxide to balance pH which improves immunitythere are some websites that are against chlorine dioxide but many that show that it works, so it is a matter of perspective what you believe [paraphrased] (Electrician 4)
Priority testing gets them back to work	But yeah, there hasn't been much else as far as support, like where we could get tested, information on how we get tested. (EMT 1) I didn't want anybody [in law enforcement] to get sick at work from it, and I didn't want community members to get sick from my teams. So I would just err and put them off from work until we heard back, but the priority testing is really the only option I have [to get them back to the field] (Police 2)	[My employer] didnt give us a place to get tested I wish that we had more access to testing for drivers We drive every day, for 5 days a week and we take care of all kinds of people (Bus Driver 5)
Fear of bringing an amorphous enemy home	[Police officers] have a lot of anxiety about bringing it home to their families and that sort of thing. (PoliceMD 3) The thing that obviously is concerning to people is the last thing we want to do is bring this thing home, right? Especially those of us who have small kids and spouses. (Police 4)	Its been very tough. I need to work even though I dont feel comfortable doing so (Grocery 4) At first, I couldnt breathe. The first two weeks, I was panicked. What happens if I die? I stopped reading the news, and it's getting much better now. (Gas 1)
Lack of resources to fight the pandemic	I think what we're having that's been a disappointment is the lack of PPEs^c^. And then the people that have been price gouging with the PPEs And then we get them, and they're not N95s. So we can't use them in the field. (EMT 6) I mean our city fire departments are getting [mixed] messages of you're heroes, but the city's not going to make nearly as much money this year as they normally do, so we're going to slash your budget. (FireMD 2)	We have coworkers that already died, we have coworkers they already have COVID. Now they give us a set of masks, a little thin piece of material. Come on. (Bus Driver 5)
Need for interprofessional coordination (first responders only)	And I can say that we've [transported] 13 positive COVID cases. I've only been notified on two of them. (EMT/Fire 5) Where to park, where to go, what the entrance point is, what's the exit point [at the hospital], is there a priority line? If law enforcement comes in, and they need to do something, do they have to wait in the general line and wait behind 10 people, or are they going to be let in a different way or checked in quickly, or given priority? (Police 4)	Did not emerge in this group
Double-edged culture of heroism (first responders only)	There's a certain expectation that we hold ourselves to when we come to work and put a uniform on. Regardless of the environment or whatever's presentedwe just do our best to protect ourselves. Are some of us fearful of it [COVID-19]? Sure, but it doesn't create necessarily anxiety. (Police 5) I think that because [police officers] tend to be the typical first responders, they hold things in so they don't deal with anxiety about these sorts of issuesThey only reach a crisis point before they admit it, is what tends to happen. (PoliceMD 3) I think that it was tricky because I felt like we were testing pretty much anybody we wanted to at Stanford if we had a suspicion, but it was harder to tell a firefighter, Hey, you probably should get tested. You had a high risk exposure. (FireMD 1)	Did not emerge in this group

^a^EMS: emergency medical services*.*

^b^EMT: emergency medical technician.

^c^PPE: personal protective equipment.

#### Need for Personalized Answers With No Clear Playbook

First responders and essential workers reported that throughout the pandemic, they found themselves adjusting to new protocols and professional responsibilities, which came with many uncertainties.

First responders wanted to know how standard work protocols should change in the setting of the COVID-19 pandemic, particularly in the setting of limited personal protective equipment (PPE) resources (FireMD 1). These protocol questions impacted standard procedures, such as transporting evidence from an out-of-state crime scene involving suspects with confirmed COVID-19 (PoliceMD 5), as well as human resource and personal health concerns, such as contemplating how to manage COVID-19 risk for pregnant staff and officers (Police 3). In the absence of any precedent, professionals were forced to make challenging decisions with limited information.

Essential workers also wanted personalized information (Gas 2, Electrician 4, Gas 1), though these needs were not typically related to altered professional responsibilities but were instead related to language, personal preferences, and need for increased financial support. Both groups wanted more local dynamic information about COVID-19positive cases (Gas 3, Police 4, EMT/Fire 5).

#### Parsing Information Takes Time

Members of both groups acknowledged an overabundance of COVID-19 information through the media, internet, social media, and their professional settings. This aspect was particularly challenging given the dynamic nature of the available evidence (EMT/Fire 3, Police 2). Although the first responders appreciated county-level dashboards, professional daily emails, and Incident Command Center communications that made this information easier to digest (Fire/MD 4, EMT/Fire 5, Police 3, Police 5), others acknowledged significant time spent by either themselves (Gas 2, Grocery 3, Police 1) or someone in their work setting to digest the information (EMT/Fire 7).

Although first responders received their information largely from professional networks, essential worker information sources leaned toward social media (Electrician 4) and other websites (Grocery 2). The quantity of data led to skepticism:

Is there any [accurate data]? I feel so in the blind. I feel like there's information flying from every direction and so much of it is unreliable.

EMT/Fire 7

#### Misinformed Beliefs Fuel Conflict

The lack of reliable data sources seemed to fuel mistrust and misinformation. Both groups expressed an explicit distrust of media (EMT 4, Bus Driver 5) and government (Food Services 5). Workers also reported encountering community members through their work who were misinformed, many of whom were reported to believe that the pandemic was a conspiracy (EMT/Fire 3). Lapses in community cooperation with mask requirements created novel conflict on the job, including the need to address hostile behavior in the community (Gas 1, EMT/Fire 3).

#### Priority Testing Gets Them Safely Back to Work

Both groups acknowledged some challenges in scheduling testing both through the app and in general through a work referral or on their own. Once a test was scheduled, however, experiences were positive. Interviewees emphasized that prioritizing testing was critical for enabling first responders and essential workers to return to their duties. In first responder organizations in which testing access was straightforward, this was thought to be due to designated COVID-19 support within the organization (Police 5, Electrician 4, EMT/Fire 5).

By contrast, essential workers reported frustration with accessing testing (Bus Driver 5). In the majority of cases where testing support was not available through work, participants sought alternate options through their primary care providers or the county (Bus Driver 5, Grocery 4, Gas 2); however, 1 participant was unsure of how to access testing at all (Gas 3).

#### Fear of Bringing an Amorphous Enemy Home

The stressful effects of the pandemic were salient across several interviews in both groups. These concerns centered on the amorphous nature of the disease (EMT/Fire 2), a need to go to work despite not feeling safe (Grocery 4), and fear of bringing the virus home to family (PoliceMD 3, EMT 4, Grocery 4, Food Services 5). Early high mortality rates documented in other countries contributed to widespread paranoia (EMT/Fire 5, Electrician 4), and reading the news worsened anxiety for some (Gas 1, Grocery 1).

The nature of a pandemic also meant that the workers families were affected, demanding their attention on top of their existing professional duties (FireMD 4). The prolonged and unpredictable nature of the pandemic was described as a roller coaster (EMT/Fire 7) with a hurry up and wait pace (EMT/Fire 2). One responder had never been so scared and so bored in my life (EMT/Fire 2). The mental suffering that resulted from the COVID-19 pandemic impacted both first responder and essential worker communities, although open discussions related to mental health were described as muted (Bus Driver 5). Although no first responders acknowledged wanting additional mental health support (see Culture of Heroism below), at least one essential worker acknowledged she did not have the support she needed (Food Services 5).

#### Lack of Resources to Fight the Pandemic

A lack of resources emerged as a dominant theme in conversations for both first responders and essential workers. Several participants noted insufficient quantities of PPE, to the point that police officers were reported to go begging at one point to obtain a sufficient supply (PoliceMD 3). Public budget cuts and subsequent staffing shortages were also cited as major challenges (Police 4). Although some workers belonged to unions, which made some efforts to support workers, their impact was perceived to be limited (Bus Driver 5, Grocery 4).

The broader economic impact of the pandemic also shifted the workers financial situations. Although some felt that the essential nature of their jobs made them more secure (Grocery 1, 2), this sentiment did not extend to everyone, as essential workers reported being furloughed (Bus Driver 5) or felt insecure in their position (Gas 1, 2). Finding financial support in the case of illness was a major challenge (EMT 1), particularly for workers who were new or did not have sick days (EMT/Fire 7). For this reason, some cited a reluctance to get tested because they were unable to take two weeks off work (EMT 6). Essential workers, more than first responders, cited financial insecurity as a major ongoing challenge (Grocery 4, Food Services 5).

#### Need for Interprofessional Coordination (First Responders Only)

Some themes emerged that were specific to first responders, including the need for interprofessional coordination. Health system support of first responders was felt to be critical, given their role as the tip of the spear (EMT/Fire 5). Specific areas for coordination with health systems included follow-up information regarding a transported patients health status. When these communication channels fail, anxiety increases significantly, as first responders wonder about potential exposure (EMT/Fire 5).

Another challenge emerged relating to hospital entry to respond to a crisis while bypassing the COVID-19 fever checkpoint (Police 4). In this case, the participant pointed out that because the other professional organizations already screen their workers before they start their shifts, the health system should be able to skip this step. Finally, coordination of COVID-19 information across health systems and counties up the Incident Command System was also cited as a challenge (Police 3, FireMD 4, PoliceMD 5).

#### Double-Edged Culture of Heroism (First Responders Only)

Several first responders shared their ongoing willingness to face the pandemic despite unfavorable circumstances because they had made a commitment to serve when choosing their profession (EMT 1, 4; EMT/Fire 3, 14; Police 1, 4, 5). Some described an underlying desire to be helpful (EMT/Fire 2) and an expansive view of their own roles in the community (Police 4). The role of choice in their profession was reported to protect against the adverse mental health effects of their roles, as with the attitude that this is what I signed up for (EMT/Fire 3).

However, this cavalier culture also led to a reluctance to report symptoms and obtain health services (PoliceMD 3). Even physician advisors shared reluctance to tell first responders they needed testing (FireMD 1). One EMT/Fire responder noted that few first responders recognized that their own colleagues may be struggling and undergoing therapy to handle their mental stress (EMT/Fire 2).

### Solutions to Challenges in the Field

Participants provided several solutions to address these challenges, some of which they thought could be delivered through an app (Table 3). To address misinformation and fear of SARS-CoV-2, several participants validated the potential benefit of succinct, accurate information delivered through an app. Others thought that sharing accurate information through social media would be most effective given the increased visibility of these platforms among essential workers. Yet others felt the need for higher touch strategies, possibly including holding local question and answer (Q&A) sessions (FireMD 2). The personalization of information in any solution was a recurrent theme:

So to me that [personalized information] seems more in line with our overall philosophy at Stanford of precision medicine. Its more like precision information because then its filtered, its more specific to you. You dont get confused when youre reading about stuff that doesnt apply to you.

FireMD 4

In the absence of precision information, many solutions were developed in the field: Some of the crews just kind of came up with stuff on their own (FireMD 4). Other essential workers found support in managing conflict with the public from their employers (Grocery 4) or through their own volition. One bus driver reported company instructions to call a hotline when riders were not compliant with wearing masks, but she instead refused to drive the bus: I use my [face covering gesture] until they put it on (Bus Driver 5).

Expanding the physician-first responder partnerships to a broader audience, particularly to the police, where only informal relationships existed, was suggested. A specific request was for a designated number or person within the health system to call when the COVID-19 status of a transported patient was under question.

Personalized information also encompassed health systemspecific COVID-19related information. Some first responders expressed a desire for health systems to be forthcoming about their own infection control practices and current rates: Thats where you just put the notion of, Hey, were [health system] safeYou can transport patients to our hospitals and we wont get you contracted (EMT 4). First responders also wanted detailed, localized COVID-19positivity data, expressing a desire to better understand the geographic spread of cases throughout the county (Police 4, EMT/Fire 2, 7).

**Table 3:** Participant-derived digital and nondigital solutions to support first responders and essential workers during a pandemic.

Challenge	Participant-derived digital and nondigital solutions	Example quotations
Need for personalized answers with no clear playbook	Physicianfirst responder department partnerships (Fire MD 1, 4, Police MD 3, 5, Police 1, 3, 5, EMT^a^/Fire 7) Physician-led Q&A^b^ sessions (Fire MD 2) Colocation of county officer (Police 2)	[E]specially in our early stages of our Incident Command Center, we created an entire team called a Virus Response Team at the end of the day they're just your everyday cop I think it would have been helpful to have guidance from somebody a little bit more knowledgeable in the field versus just picking up the information from the distributor and figuring it out from there. (Police 3)
Parsing information takes time/misinformed beliefs fuel conflict	Physicians coming into work huddles to provide relevant, accurate COVID-19 information (Grocery 2, 4, Police 5) Provide information on an app (Grocery 1, 4, Gas 1, EMT/Fire 2, 3, EMT 4, Police 4, Electrician 4, Food Services 5) Provide information and/or advertise app on social media (Electrician 4), telenovelas (Food Services 5)	Maybe coming and visiting. There are two huddles a daymaybe you [health workers] could pop in during those. (Grocery Worker 2) I think that's a large part of my role is that for the most part, the personnel in my departments know me and so if they ask me a question and I answer a question, or if I bring something up, they basically trust most of what I say. Or at least have enough kind of history with me that they at least need to think about it... So, I mean part of my role is to be kind of a sounding board for what's true and what's probably not true (FireMD 2)
Priority testing gets them back to work	Provide comprehensive information on testing resources in the county (Police 1, FireMD 4) Provide priority testing for first responders and essential workers (Police 1, 4, 5; Police MD 3)	I think just a formalized priority testing system for law enforcement has a huge impact on the organization these are the essential workers that if they're not at work or they are sick, it affects teams of people, and cars, and community contact. (Police 1)
Fear of bringing an amorphous enemy home	Provide resources for first responders to help them manage stress and anxiety (Police MD 3) Ask the public to share whether someone is sick in the presence of emergency personnel (EMT/Fire 5)	[M]aking sure you've got reliable sources of information and things like that [can be helpful]because it can just help identify that yes, you're having anxiety about this. Here's some tools to help with it. (PoliceMD 3)
A lack of resources to fight the pandemic	Share PPE^c^ and other resources when possible (EMT 4, 6) Promote consistent mask use to the public (EMT/Fire 7, 3)	I guess just shortages on masksIt's our company that was supposed to provide for us, but it's even hard for us to get supplies sometimes. (EMT 4) Wearing masks and not getting other people sick. That's kind of supporting us... I've been telling people that have been asking me the best thing they can do is to keep themselves healthy, to not over impact our system by, all the things that everyone's being asked to do by washing their hands and covering their face when they're in public. That's certainly a factor. (EMT/Fire 7)
Need for interprofessional coordination (first responders only)	Keep first responders updated on the outcomes of patients under investigation (EMT 6, EMT/Fire 5, 7) Keep first responders informed on the process of entering hospitals to facilitate efficient emergency response (Police 4)	Hospitals should have their infectious disease control departments, and they should be responsible for reaching out to people when they make positive COVID results. (EMT 6)
A double-edged culture of heroism (first responders only)	Physicianfirst responder department partnerships (PoliceMD 3) Provide resources for first responders to help them manage stress and anxiety (Police MD 3)	They're not that kind of group that usually shares those things, unless you have a preexisting relationship with them. That can be hard to get to. (PoliceMD 3)

^a^EMT: emergency medical technician.

^b^Q&A: question and answer.

^c^PPE: personal protective equipment.

### Heath Worker Partnerships at the Heart of a Community Solution

A number of participants desired a stronger connection to a health worker during COVID-19. The physician partners themselves felt they were often the best resource to support first responders during the pandemic: we are the best conduit [through] the already established trust that we've built up through the years (FireMD 1). These participants felt their role encompassed the development and maintenance of long-term, trusting relationships with these first responder communities (FireMD 1, PoliceMD 5). First responders corroborated the special nature of the relationship in times of crisis, as one physician partner was described as a really good resource. He's been coming by the station now too, to talk with us and to check in on COVID related topics, as well as other [topics] (EMT/Fire 7).

In addition to their formal physician partnerships, participants also received information through informal networks into the health care community, such as a former employee who worked in the health system at the time of the interview (Grocery 3). Those with shift-based work welcomed the idea of a health worker presence at the beginning of their shift: That may be an opportunityto have someone present during those briefings to talk on any new developments or anything COVID related (Police 5, Grocery 2). The human, in-person element of this relationship was emphasized.

## Discussion

### Principal Findings

Digital initiatives such as the Stanford COVID-19 Guide App have the potential to equip first responders and other essential workers with accurate information and access to testing resources, although adoption may depend on targeted publicity and early involvement of stakeholders with pre-existing relationships within these communities. While first responders are often included under the broader category of essential workers during outreach efforts, our findings suggest they face both overlapping challengesrelated to the need for personalized and accurate information, access to testing, and ongoing personal safetyand divergent challenges, such as the need for interprofessional coordination and a double-edged culture of heroism, seen in the first responder sample alone. Optimal strategies for supporting each group should therefore differ based on their divergent needs, channels for outreach, appetite for digital and relationship-based approaches, and relationship to the health system (Table 4).

Physician partnerships emerged as a key resource for first responder departments, and these relationships are recognized as a foundational underpinning of a pandemic response [52-54]. Formalizing and nourishing these relationships holds promise in strengthening the overall ability of the health system to support its community during a pandemic. Interprofessional coordination was also cited as a major challenge, hampered by interoperability challenges between the electronic health records of EMS and the receiving health system [55]. Given these challenges, a trusted point of contact (eg, emergency department nurse line) between EMS and the health system may be explored as an alternate solution in a pandemic setting.

Our findings also suggest that first responders see increased risk as an integral part of assuming their role and may therefore adapt more readily to a new pandemic. Conversely, nonfirst responder essential workers decidedly *do not* see their roles as inherently risky outside a pandemic scenario. Without the training and cultural reinforcement around managing increased risk as a part of standard work, essential workers may suffer disproportionately from the onset of an infectious disease pandemic. The contributions of these workers during a pandemic should therefore not be diminished, and prioritized health services and hazard pay may be justified [56].

Similarly, misinformation emerged as a widespread challenge among the broader community. Although first responders have at least a basic degree of medical training and serve as a source of information to laypersons during a pandemic [57], essential workers often lack this formal training. Health literacy, defined as the ability to acquire, understand and enlist health information [58] becomes essential during a pandemic as first responders and essential workers come into regular, direct contact with members of the community. The reported reliance of essential workers on social media for information is concerning given its link to the spread of unvetted information [59-61]. Efforts to build health literacy, not only through digital interventions but also through channels with widespread use among vulnerable populations, including essential workers and non-English speakers, are urgently needed.

We note that the early learnings described here must be interpreted with the context that the COVID Guide App was rapidly deployed in just under three months after the first reported COVID-19 case in the United States [62]. Balancing the accuracy of an intervention in achieving its aim with the goal of rapid deployment will no doubt remain a challenge during this pandemic and other disasters; we recommend a standing advisory board with representation from target populations that can be mobilized in the short term to inform intervention development. In the long-term, community-based participatory research and human-centered design are parallel and overlapping methodologies that emphasize user expertise and creative problem-solving, with documented success in vulnerable populations [63-65]. These tools may inform improved evolutions of community interventions.

**Table 4:** Digital and nondigital recommendations to support first responders and essential workers during a pandemic.

Consideration	Recommendations	
	First responders	Essential workers
High need areas	Advice on work adaptation Streamlined accurate and timely information Interprofessional coordination around follow up of patient diagnostic status and logistics Priority access to rapid testing Steady access to PPE^a^	Acceptable resources to access accurate and timely information Priority access to rapid testing Steady access to PPE Assistance navigating financial resources
Primary channels for outreach	Formal physician partnerships with first responder departments Employers (eg, through county infection control officers)	Employers Social media Telenovelas and other culture-specific channels
Role of an app-based intervention	Small role Provide nonpublic up-to-date information on working with health systems directly (eg, priority building entrances, rapid testing schedule, contact information to receive outcome updates on patients under investigation)	Large role Source of credible health information that builds health literacy (eg, topics such as protecting yourself and your family, viral transmission, symptom screening)
Role of physician/health worker outreach	Large role Provide guidance on safe work adaptations Facilitate provision of rapid testing and follow-up medical support Assist in the dissemination of relevant health information	Small role Provide guidance on safe work adaptations Present health information to local businesses
Role of local health system	Large role Facilitate prioritized access to testing and follow-up health services Provide follow-up information to emergency medical service workers regarding patient diagnostic status Develop and disseminate streamlined protocols for entering and exiting healthcare buildings during a pandemic	Large role Facilitate prioritized access to testing and follow-up health services Directly address misinformation related to pandemic

^a^PPE: personal protective equipment.

### Limitations and Future Work

Limitations of this study include a lack of systematic sampling across professions in the nonfirst responder essential worker population, which resulted from challenges accessing this group in the field. We are unaware of similar work reaching this vulnerable population, which suggests this sample is still of value. Further, Spanish-speakers predominated our nonEnglish-speaking sample, which reflects local demographics but does not account for the full variety of languages spoken in the community [43]. Furthermore, qualitative data capture differed between the first responders (verbatim transcriptions) and essential workers (near-verbatim field notes). This decision was made to protect essential worker anonymity given their potentially vulnerable position near their place of work. Efforts to mitigate data loss from an absence of recordings included a dedicated note-taker and immediate debrief discussions between two or more researchers to expand data capture. Finally, our learnings are limited to our local community, as the Bay Area may not be representative of populations and resources available elsewhere.

Future work will benefit from increased attention to the variety of first languages that exist within the community, including non-Spanish languages. The incorporation of a mental health component to this digital solution, reported as a need here and evaluated in other settings [66,67], may also be an opportunity for future work. Finally, evaluating digital support systems for EMS personnel as they face an increased number of deaths in the field as a result of the pandemic [68,69]supporting decisions related to triage and/or end of life careis another important area for future research.

### Conclusion

First responders and essential workers face shared challenges related to obtaining accurate information and testing services, and securing their personal safety. Digital interventions such as mobile applications have the potential to combat these challenges through the provision disease-specific information and access to testing services. Such solutions are likely to be most effective if delivered as part of a larger ecosystem of support, and with early and direct input from those in these professions to understand how best to meet their specific needs. Given varying challenges between first responders and non-first responder essential workers, our results indicate that differentiated interventions may leverage shared insights while also acknowledging differences in their occupational requirements and culture.

## Appendix

Multimedia Appendix 1 First responder and essential worker semistructured interview protocols.

## Abbreviations

EMS: emergency medical services
EMT: emergency medical technician
PPE: personal protective equipment
Q&A: question and answer
SARS: severe acute respiratory syndrome
SHC: Stanford Health Care

## References

[1] Awais SB, Martins RS, Khan MS (2021). Paramedics in pandemics: protecting the mental wellness of those behind enemy lines. Br J Psychiatry.

[2] Djalante R, Shaw R, DeWit A (2020). Building resilience against biological hazards and pandemics: COVID-19 and its implications for the Sendai Framework. Prog Disaster Sci.

[3] Bricker L, Petermann T, Hines M, Sands J (2013). The Legal Definitions of a First Responder. Transportation Research Board.

[4] The Dialogue: A Quarterly Technical Assistance Journal on Disaster Behavioral Healthffects of Trauma on First Responders. Substance Abuse and Mental Health Services Administration.

[5] Bai Y, Lin C, Lin C, Chen J, Chue C, Chou P (2004). Survey of stress reactions among health care workers involved with the SARS outbreak. Psychiatr Serv.

[6] Styra R, Hawryluck L, Robinson S, Kasapinovic S, Fones C, Gold WL (2008). Impact on health care workers employed in high-risk areas during the Toronto SARS outbreak. J Psychosom Res.

[7] Paterlini M (2020). On the front lines of coronavirus: the Italian response to covid-19. BMJ.

[8] Du J, Dong L, Wang T, Yuan C, Fu R, Zhang L, Liu B, Zhang M, Yin Y, Qin J, Bouey J, Zhao M, Li X (2020). Psychological symptoms among frontline healthcare workers during COVID-19 outbreak in Wuhan. Gen Hosp Psychiatry.

[9] Lai J, Ma S, Wang Y, Cai Z, Hu J, Wei N, Wu J, Du H, Chen T, Li R, Tan H, Kang L, Yao L, Huang M, Wang H, Wang G, Liu Z, Hu S (2020). Factors associated with mental health outcomes among health care workers exposed to coronavirus disease 2019. JAMA Netw Open.

[10] DePierro J, Lowe S, Katz C (2020). Lessons learned from 9/11: mental health perspectives on the COVID-19 pandemic. Psychiatry Res.

[11] Stogner J, Miller BL, McLean K (2020). Police stress, mental health, and resiliency during the COVID-19 pandemic. Am J Crim Justice.

[12] Gershon RRM, Magda LA, Qureshi KA, Riley HEM, Scanlon E, Carney MT, Richards RJ, Sherman MF (2010). Factors associated with the ability and willingness of essential workers to report to duty during a pandemic. J Occup Environ Med.

[13] Garrett AL, Park YS, Redlener I (2013). Mitigating absenteeism in hospital workers during a pandemic. Disaster Med Public Health Prep.

[14] Weber L, Hilfinger Messias DK (2012). Mississippi front-line recovery work after Hurricane Katrina: an analysis of the intersections of gender, race, and class in advocacy, power relations, and health. Soc Sci Med.

[15] Maurer LR, Perez NP, Witt EE, Ortega G (2020). Protecting our own: equity for employees as hospitals battle COVID-19. Health Equity.

[16] Chersich MF, Gray G, Fairlie L, Eichbaum Q, Mayhew S, Allwood B, English R, Scorgie F, Luchters S, Simpson G, Haghighi MM, Pham MD, Rees H (2020). COVID-19 in Africa: care and protection for frontline healthcare workers. Global Health.

[17] The Lancet (2020). The plight of essential workers during the COVID-19 pandemic. Lancet.

[18] Benhamou K, Piedra A (2020). CBT-informed interventions for essential workers during the COVID-19 pandemic. J Contemp Psychother.

[19] De BR, Balanz- MV, Mota J Depression, anxiety and lifestyle among essential workers: a websurvey from Brazil and Spain during the COVID-19 pandemic. JMIR Preprints..

[20] McKay D, Asmundson GJ (2020). Substance use and abuse associated with the behavioral immune system during COVID-19: the special case of healthcare workers and essential workers. Addict Behav.

[21] McCormack G, Avery C, Spitzer AK, Chandra A (2020). Economic vulnerability of households with essential workers. JAMA.

[22] Rogers TN, Rogers CR, VanSant-Webb Elizabeth, Gu LY, Yan B, Qeadan F (2020). Racial disparities in COVID-19 mortality among essential workers in the United States. World Med Health Policy.

[23] Saultz J (2020). Essential workers. Fam Med.

[24] Ho CS, Chee CY, Ho RC (2020). Mental health strategies to combat the psychological impact of CoronavirusDisease 2019 (COVID-19) beyond paranoia and panic. Ann Acad Med Singap.

[25] Maunder R (2004). The experience of the 2003 SARS outbreak as a traumatic stress among frontline healthcare workers in Toronto: lessons learned. Philos Trans R Soc Lond B Biol Sci.

[26] Iacobucci G (2020). Covid-19: all essential workers in England can now be tested. BMJ.

[27] White DB, Lo B (2020). A framework for rationing ventilators and critical care beds during the COVID-19 pandemic. JAMA.

[28] Dennerlein JT, Burke L, Sabbath EL, Williams JAR, Peters SE, Wallace L, Karapanos M, Sorensen G (2020). An integrative total worker health framework for keeping workers safe and healthy during the COVID-19 pandemic. Hum Factors.

[29] Farcas A, Ko J, Chan J, Malik S, Nono L, Chiampas G (2020). Use of incident command system for disaster preparedness: a model for an emergency department COVID-19 response. Disaster Med Public Health Prep.

[30] Lurie N, Fremont Allen (2009). Building bridges between medical care and public health. JAMA.

[31] Kringos D, Carinci F, Barbazza E, Bos V, Gilmore K, Groene O, Gulcsi L, Ivankovic D, Jansen T, Johnsen SP, de Lusignan S, Mainz J, Nuti S, Klazinga N, HealthPros Network (2020). Managing COVID-19 within and across health systems: why we need performance intelligence to coordinate a global response. Health Res Policy Syst.

[32] (2020). Digital technology for COVID-19 response. World Health Organization.

[33] Drew David A, Nguyen Long H, Steves Claire J, Menni Cristina, Freydin Maxim, Varsavsky Thomas, Sudre Carole H, Cardoso M Jorge, Ourselin Sebastien, Wolf Jonathan, Spector Tim D, Chan Andrew T, COPE Consortium (2020). Rapid implementation of mobile technology for real-time epidemiology of COVID-19. Science.

[34] Zamberg I, Windisch O, Agoritsas T, Nendaz M, Savoldelli G, Schiffer E (2020). A mobile medical knowledge dissemination platform (HeadToToe): mixed methods study. JMIR Med Educ.

[35] Proctor EK, Landsverk J, Aarons G, Chambers D, Glisson C, Mittman B (2009). Implementation research in mental health services: an emerging science with conceptual, methodological, and training challenges. Adm Policy Ment Health.

[36] O'Cathain A, Murphy E, Nicholl J (2010). Three techniques for integrating data in mixed methods studies. BMJ.

[37] Fetters MD, Curry LA, Creswell JW (2013). Achieving integration in mixed methods designs-principles and practices. Health Serv Res.

[38] Stanford M Stanford Medicine COVID-19 Guide for First Responders and Essential Workers. Stanford Medicine.

[39] Hendersen J, McCullough E, Treuhaft S (2020). A profile of frontline workers in the Bay Area. Bay Area Equity Atlas.

[40] (2019). American Community Survey 2014-2018 5-year estimates. United States Census Bureau.

[41] Hilal S, Jones D (2014). A package deal: police, fire, and EMS all in one. The Police Chief.

[42] Emergency Medical Services Fellowship. Stanford Medicine.

[43] San Francisco Bay Area. Bay Area Census.

[44] Damschroder LJ, Aron DC, Keith RE, Kirsh SR, Alexander JA, Lowery JC (2009). Fostering implementation of health services research findings into practice: a consolidated framework for advancing implementation science. Implement Sci.

[45] Rev.

[46] Beebe J (1995). Basic concepts and techniques of rapid appraisal. Hum Organ.

[47] Brown-Johnson CG, Chan GK, Winget M, Shaw JG, Patton K, Hussain R, Olayiwola JN, Chang S, Mahoney M (2019). Primary Care 2.0: design of a transformational team-based practice model to meet the quadruple aim. Am J Med Qual.

[48] Gale RC, Wu J, Erhardt T, Bounthavong M, Reardon CM, Damschroder LJ, Midboe AM (2019). Comparison of rapid vs in-depth qualitative analytic methods from a process evaluation of academic detailing in the Veterans Health Administration. Implement Sci.

[49] Miles M, Huberman A, Saldana J (2019). Qualitative Data Analysis, 4th edition.

[50] Proctor E, Silmere H, Raghavan R, Hovmand P, Aarons G, Bunger A, Griffey R, Hensley M (2011). Outcomes for implementation research: conceptual distinctions, measurement challenges, and research agenda. Adm Policy Ment Health.

[51] Brown-Johnson Cati, Safaeinili N, Zionts D, Holdsworth LM, Shaw JG, Asch SM, Mahoney M, Winget M (2020). The Stanford Lightning Report Method: a comparison of rapid qualitative synthesis results across four implementation evaluations. Learn Health Syst.

[52] Digregorio H, Graber JS, Saylor J, Ness M (2019). Assessment of interprofessional collaboration before and after a simulated disaster drill experience. Nurse Educ Today.

[53] Barnett-Vanes A, Luis Guinto RL (2013). Disaster curricula in medical and health care education: adopting an interprofessional approach. Prehosp Disaster Med.

[54] Willems A, Waxman B, Bacon AK, Smith J, Peller J, Kitto S (2013). Interprofessional non-technical skills for surgeons in disaster response: a qualitative study of the Australian perspective. J Interprof Care.

[55] Sexton M, Orchard C (2016). Understanding healthcare professionals' self-efficacy to resolve interprofessional conflict. J Interprof Care.

[56] Hecker S (2020). Hazard pay for COVID-19? Yes, but it's not a substitute for a living wage and enforceable worker protections. New Solut.

[57] Roberts A, Nimegeer A, Farmer J, Heaney DJ (2014). The experience of community first responders in co-producing rural health care: in the liminal gap between citizen and professional. BMC Health Serv Res.

[58] Paakkari L, Okan O (2020). COVID-19: health literacy is an underestimated problem. Lancet Public Health.

[59] Zarocostas J (2020). How to fight an infodemic. Lancet.

[60] Bursztyn L, Rao A, Roth C, Yanagizawa-Drott D (2020). Misinformation during a pandemic. SSRN Journal.

[61] Vinck P, Pham PN, Bindu KK, Bedford J, Nilles EJ (2019). Institutional trust and misinformation in the response to the 201819 Ebola outbreak in North Kivu, DR Congo: a population-based survey. Lancet Infect Dis.

[62] Holshue ML, DeBolt C, Lindquist S, Lofy KH, Wiesman J, Bruce H, Spitters C, Ericson K, Wilkerson S, Tural A, Diaz G, Cohn A, Fox L, Patel A, Gerber SI, Kim L, Tong S, Lu X, Lindstrom S, Pallansch MA, Weldon WC, Biggs HM, Uyeki TM, Pillai SK (2020). First case of 2019 novel coronavirus in the United States. N Engl J Med.

[63] Chen E, Leos C, Kowitt SD, Moracco KE (2020). Enhancing community-based participatory research through human-centered design strategies. Health Promot Pract.

[64] Kia-Keating M, Santacrose DE, Liu SR, Adams J (2017). Using community-based participatory research and human-centered design to address violence-related health disparities among Latino/a youth. Fam Community Health.

[65] Durand M, Alam S, Grande SW, Elwyn G (2016). 'Much clearer with pictures': using community-based participatory research to design and test a Picture Option Grid for underserved patients with breast cancer. BMJ Open.

[66] Gratzer D, Strudwick G, Yeung A (2019). Mental illness: is there an app for that?. Fam Syst Health.

[67] Lee RA, Jung ME (2018). Evaluation of an mHealth app (DeStressify) on university students' mental health: pilot trial. JMIR Ment Health.

[68] Lerner E, Newgard C, Mann N (2020). Effect of the coronavirus disease 2019 (COVID-19) pandemic on the U.S. emergency medical services system: a preliminary report. Acad Emerg Med.

[69] Friedman J, Caldern-Villarreal A, Bojorquez L, Hernndez C, Schriger D, Hirashima E Excess out-of-hospital mortality and declining oxygen saturation: the sentinel role of EMS data in the COVID-19 crisis in Tijuana, Mexico. medRxiv..

