# Supplementary material for: An App-Based Intervention to Support First Responders and Essential Workers During the COVID-19 Pandemic: Needs Assessment and Mixed Methods Implementation Study
Source: J Med Internet Res. 2021 May 20;23(5):e26573. doi: 10.2196/26573 (PMC8139393; doi:10.2196/26573)
Supplement: Multimedia Appendix 1 [file jmir_v23i5e26573_app1.docx]

**APPENDIX**

**Appendix A. First Responder and Essential Worker Semi-Structured Interview Protocol**

| Questions |
| --- |
| 1. What is your role as a first responder?  - What is your favorite part of your job? |
| 2. Tell me about your experience so far as a first responder during COVID-19.  - What parts surprised you?  - What parts disappointed you? |
| 3. What is your *community* doing to support you in your role as a first responder? What else could they do to support you?  - What has your experience been with Stanford Health Care? Has SHC or any other health system (collection of hospitals and clinics) in the area done anything to support you during this time? What else could they do? |
| 4. Do you know how you might currently obtain services related to COVID-19, such as obtaining a diagnostic test?  - What does this process look like?  - What about the serology antibody test?  - How do you know whether you need a test?  - Have you used a symptom checker? Is it useful? |
| 5. How do you currently obtain accurate and up to date information related to COVID-19?  - What information are you looking for? What information on COVID-19 do you need to do your job?  - Is there a process in your organization to obtain reliable COVID-19 information?  - How trustworthy do you think these sources are?  - Now that there is so much more information on COVID-19 available, what information are you still looking for? |
| 6. What do you know about the Stanford Health Care frontline responder app? [If no experience, describe the app and skip to relevant questions.]  - Where did you first hear about it?  - Please tell me about your experience, if any, in using this app.  - How could the app be improved?  - Do you typically use apps to support you in your job?  - What would a useful COVID-19 app for first responders look like in your opinion? |
| 7. How afraid are you of contracting COVID-19?  - Some people report negative emotions to responding to pandemics. How relatable does this feel to you?  - What might the community do to help you or other frontline responders with similar challenges? |
